# 15,16-dihydrotanshinone I inhibits EOMA cells proliferation by interfering in posttranscriptional processing of hypoxia-inducible factor 1

**DOI:** 10.7150/ijms.60774

**Published:** 2021-07-11

**Authors:** Peiwen Duan, Yingying Huang, Kai Chen, Cheng Cheng, Zhixiang Wu, Yeming Wu

**Affiliations:** 1 Department of Pediatric Surgery, Xinhua Hospital, School of Medicine, Shanghai Jiaotong University, Shanghai, China; 2 Division of Pediatric Oncology, Shanghai Institute of Pediatric Research, Shanghai, China; 3 Department of Reconstructive Surgery, Xinhua Hospital, School of Medicine, Shanghai Jiaotong University, Shanghai, China

## Abstract

Infantile hemangioma (IH), which threatens the physical and mental health of patients, is the most common benign tumor in infants. Previously, we found that 15,16-dihydrotanshinone I (DHTS) was significantly more effective at inhibiting hemangioma proliferation in vitro and in vivo than the first-line treatment propranolol. To investigate the underlying mechanism of DHTS, we used EOMA cells as a model to study the effect of DHTS. We compared the transcriptomes of control and DHTS-treated EOMA cells. In total, 2462 differentially expressed genes were detected between the groups. Kyoto Encyclopedia of Genes and Genomes pathway analysis revealed downregulated activity of the hypoxia-inducible factor 1 alpha (HIF-1α) signaling pathway in EOMA cells following treatment with DHTS. Thus, we investigated HIF-1α expression at protein and mRNA levels. Our results revealed that DHTS downregulated HIF-1α expression by interfering in its posttranscriptional processing, and the RNA-binding protein HuR participated in this mechanism. Our findings provide a basis for clinical transformation of DHTS and insight into pathogenic mechanisms involved in IH.

## Introduction

Infantile hemangioma (IH) is the most common soft-tissue tumor of infancy. The prevalence of IH in mature neonates is around 4.5% 1, and it increases with low birthweight and decreasing gestational age, and is as high as 23% in premature babies with birthweight lower than 1000 g 2. Most IH arises within 1-3 weeks after birth and then displays a rapid proliferation period of about 3 months (IH deep in the body of 9-12 months). During this period, approximately 12% of children have serious complications, for example, IH growing near the eyelids and nose causes permanent visual impairment and disfigurement, while IH growing deep within the body causes respiratory obstruction, nerve compression, gastrointestinal bleeding, and even be life-threatening 3.

Previously, steroids were the first-line treatment for IH. However, a study published in 2008 reported an infant with IH showing signs of improvement after receiving the β-blocker propranolol 4. After then, many centers conducted studies and published reports. Propranolol has become the first therapeutic choice for IH 5,6. However, the side effects of propranolol are inevitable. Sleep disorders, somnolence, and irritability occur in 20%-25% of patients who treated by propranolol. And about 1% of patients experience side effects such as bronchospasm or bronchiolitis and symptomatic hypotension. In addition, bradycardia, exposure to undiagnosed atrioventricular block, and hypoglycemia are rare but potentially serious side effects 7. Therefore, it is worthwhile to identify other effective and low-toxic drugs for IH.

15,16-dihydrotanshinone I (DHTS) is a natural compound found in Salvia miltiorrhiza. In our previous research, we evaluated the pharmaceutical potential of DHTS on the influence of EOMA cells growth compared with propranolol. We reported that DHTS inhibited the proliferation and angiogenesis of hemangioma more effectively than propranolol 8. Thus, exploring the mechanism of DHTS on EOMA cells is the focus of our current work.

Studies from other diseases have reported mechanisms of DHTS. For instance, DHTS was verified to exert an anti-inflammatory effect through TLR4/MyD88/NF-κB/MAPK signaling cascades in lipopolysaccharide-stimulated RAW264.7 cells 9. In addition, DHTS could reverse metabolic reprogramming in colon cancer cells through a PTEN/AKT/HIF-1α-mediated signaling pathway 10. Moreover, it was reported that DHTS could elicit therapeutic effects against metabolic syndrome. DHTS increases AMPKα phosphorylation and acetyl-CoA carboxylase phosphorylation, thus inhibiting transducer of regulated CREB activity 2 translocation, thereby promoting glucose uptake 11.

An endothelioma cell line (EOMA) originally derived from a spontaneously arising hemangioendothelioma in the 129/J mouse strain was employed as a well-established model for the study of IH 12-15. Its properties could be representative of endothelial cell biology status 16. In this study, we use EOMA cell line as a model to explore the mechanism of DHTS from the perspective of transcriptomics. Based on the results of bioinformatic analysis, we demonstrated that DHTS regulates HIF-1α expression to inhibit EOMA cells proliferation and angiogenesis and the RNA-binding protein HuR participates in this process.

## Materials and Methods

### Cell Culture and Treatment

EOMA cell line was purchased from American Type Culture Collection (Manassas, VA, USA) and cultured in 1640 medium (Gibco, Gaithersburg, MD, USA) supplemented with 10% fetal bovine serum (FBS, Gibco), 100 mg/L penicillin (Sigma, St. Louis, MO, USA), and 100 mg/mL streptomycin. Normoxic culture was kept in a humidified incubator with 5% CO_2_ and 21% O_2_ at 37 °C. The hypoxic culture was kept in a gas-controlled incubator, maintained at 1% O_2_, 94% N_2_ and 5% CO_2_ at 37 °C. Except for hypoxic treatment time, all cells were cultured in normoxic incubator. The powder of DHTS was dissolved in dimethyl sulfoxide (DMSO). Cells of DHTS-group were treated by DHTS solution and cells of control-group were treated by DMSO.

### RNA-seq Analysis

Transcriptomes of EOMA cells were investigated after 24 h DMSO or DHTS [2μM, ≤ half maximal inhibitory concentration (IC50)], including three biological replicates per group. Total RNA was extracted from EOMA cells using TRIzol Reagent (15596026, Invitrogen, USA). A total of 3 µg of RNA per sample was used as input material for RNA sample preparations. Sequencing libraries were generated using a NEBNext® UltraTM RNA Library Prep Kit (Illumina, San Diego, CA, USA) according to the manufacturer's recommendations and library quality was assessed on an Agilent Bioanalyzer 2100 system (Santa Clara, CA, USA). Sequencing was performed on Illumina HiSeq platform at Beijing Novogene (Beijing, China). Raw reads of fastq format were processed using in-house perl scripts. Hisat2 (v2.04) was used for alignment. Differential expression analysis of three biological replicates per condition was performed using cufflinks/cuffdiff (v2.2.1). Gene Ontology (GO) enrichment analysis of differentially expressed genes was implemented by the clusterProfiler R package, in which gene-length bias was corrected. GO terms with a corrected P value less than 0.05 were considered significantly enriched by differentially expressed gene (DEG) analysis. The clusterProfiler R package was used to test the statistical enrichment of DEGs in Kyoto Encyclopedia of Genes and Genomes (KEGG) pathway analysis (http://www.genome.jp/kegg/).

### Quantitative Reverse Transcription Polymerase Chain Reaction (qRT-PCR)

Total RNA was isolated with TRIzol reagent. And 1 μg of purified RNA from each sample was reverse transcribed to complementary DNA (cDNA). Real-time PCR was performed using a SYBR Green PCR Master Mix kit (Yeason, Shanghai, China), and the following primers: HIF-1α, 5ʹ-GAAACCACCCATACGTGCTTG-3ʹ (forward) and 5ʹ-AAGTCGTGCTGAATAATACCACT-3ʹ (reverse); GAPDH, 5ʹ-AAGAAGGTGGTGAAGCAGGCATC-3ʹ (forward) and 5ʹ-CGGCATCGAAGGTGGAAGAGTG-3ʹ (reverse).

### Stability of mRNA

EOMA cells were pretreated with DMSO or DHTS for 24 h. After treatment with 2 μM actinomycin D (Act D) (A1410, Sigma, USA) to inhibit* de novo* transcription, RNA of cells was extracted at three time points (0 h, 4 h, and 8 h). Analysis of kinetics for mRNA stability evaluation was carried out by quantitative PCR analysis, after extracting RNA and cDNA synthesis. Residual levels of target mRNA were calculated by comparing to total RNA at 0 h.

### Western Blot

For preparation of total cell extracts, EOMA cells were washed twice with ice-cold phosphate-buffered saline (PBS) and then lysed in RIPA lysis buffer (BioTime, Alameda, CA, USA). Protein concentration of the extract was determined by Bio-Rad protein assay (Hercules, CA, USA). Next, 20 μg protein of each extract was boiled in sodium dodecyl sulfate (SDS) buffer, subjected to SDS-polyacrylamide gel electrophoresis, and then transferred to an immobilon polyvinylidene difluoride membrane (Millipore, Burlington, MA, USA). Membranes were then blocked with 5% bovine serum albumin in Tris-buffered saline containing Tween. Primary antibodies against β-Actin, HuR, and HIF-1α (Cell Signaling Technology, Danvers, MA, USA) were used at a concentration of 1 mg/mL, followed by incubation with an alkaline phosphatase-conjugated goat anti-rabbit secondary antibody (Abcam, Cambridge, UK). Immunofluorescence was detected using a ChemiDoc acquisition instrument (Bio-Rad). ImageJ software was used to convert the image of bands into gray values for statistical analysis.

### Immunofluorescence

Cells were fixed with 4% formaldehyde for 20 min, permeabilized with 0.3% Triton X-100 for 10 min, blocked with 5% bovine serum albumin, and incubated with a primary antibody recognizing HuR (1:50) and HIF-1α (1:50). Secondary antibodies were used to detect primary antibody-antigen complexes with Cy3 (Abcam, Cambridge, UK). Nuclei were stained with 4',6-diamidino-2-phenylindole (DAPI) for 5 min in a dark room. Finally, images were detected by immunofluorescence microscopy (Leica, Wetzlar, Germany).

### RNA immunoprecipitation (RNA-IP)

To perform cross-linking of HuR and mRNA, cells were fixed in 4% formaldehyde for 10 min at room temperature. The reaction was stopped with glycine (pH 7, 0.25 M) for 5 min at room temperature. Next, cells were washed twice with ice-cold PBS and then resuspended in RIPA Buffer 1 [50 mM Tris-HCl (pH 7.5), 1% Nonidet P-40, 0.5% sodium deoxycholate, 0.05% SDS, 1 mM EDTA, 150 mM NaCl, and proteinase inhibitors] on the ice for 30 min, and ultrasonication was performed twice for 30 s each time. The lysate was centrifuged (15 min, 4°C, 12000 × g) and the supernatant was extracted. The extract was immunoprecipitated overnight at 4°C, using protein G-agarose beads preincubated with an anti-HuR or anti-igG antibody. Next, the beads were washed five times with RIPA Buffer 1 and resuspended in RIPA Buffer 2 [50 mM Tris-Cl (pH 7), 5 mM EDTA, 10 mM DTT, and 1% SDS]. Cross-linking was reversed by incubation at 70°C for 45 min. RNA was purified from immunoprecipitates with TRZol Reagent, treated with RNase-free DNase, and the total RNA was reverse transcribed. The resulting cDNA was subjected to quantitative qRT-PCR using primers against coding regions described in **qRT-PCR** section.

### siRNA Transfection

siRNA specific for HuR (5'-GGTTGAATCTGCAAAGCTTAT-3') was chemically synthesized by RiboBio (Guangzhou, China). Briefly, EOMA cells were seeded at 1 × 10^4^ cells/cm^2^ and transfected with siRNA and INTERFERin (Polyplus-Transfection SA, Illkirch, France) according to the manufacturer's protocol. The cultured medium was replaced with normal 1640 medium with FBS after 6 h. Further administration was performed after transfection for 24-48 h.

### Statistical Analysis

All experiments were repeated three times independently. Data analysis was carried out using SPSS software version 20 (IBM Corporation, Armonk, NY, USA) and GraphPad Prism 5 (GraphPad Software, La Jolla, CA, USA). The results are expressed as mean ± standard deviation. Significance was determined using a two-tailed paired Student's t test, or one-way analysis of variance (ANOVA) followed by S-N-K (S) or Dunnett's test as appropriate. p < 0.05 was considered to be statistically significant.

## Results

### Transcriptome Profiling of Control and DHTS-Treated EOMA Cells

**Figure 1:**
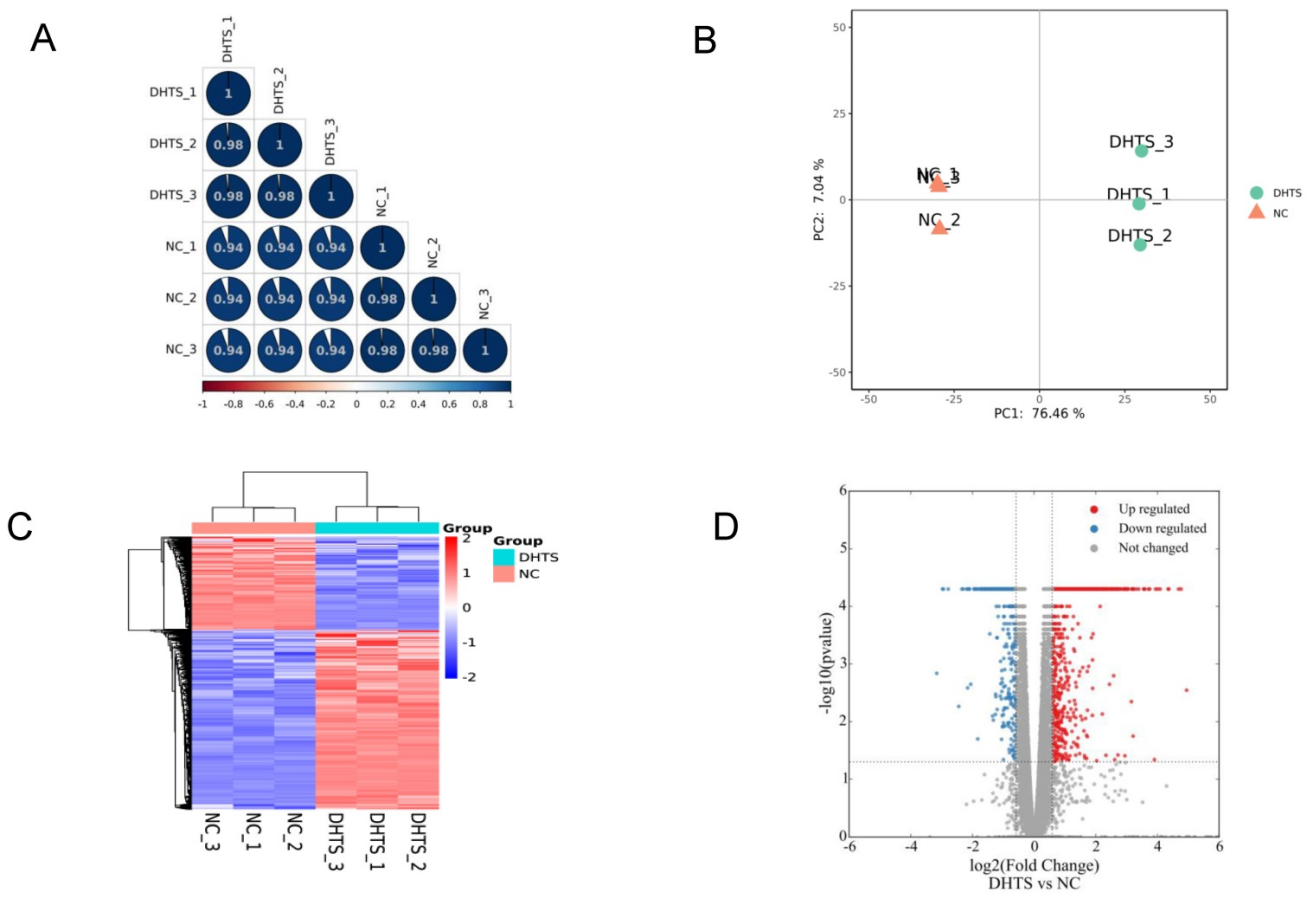
Transcriptome profiling of EOMA cells. NC group: EOMA cells treated by DMSO for 24 h; DHTS group: EOMA cells treated by 2 μM DHTS for 24 h. (**A**) Correlation analysis of biological replicates. (**B**) Principal component analysis. Each group had three biological replicates. (**C**) Hierarchical clustering of differentially expressed genes between NC group and DHTS group. (**D**) Volcano plot. X-axis represents the fold change after log2 conversion, and Y-axis represents the fold change after -log10 conversion. DEGs were acquired with the criteria of fold change > 1.5 and P < 0.05. The red points represent the upregulated genes. The blue points represent the downregulated genes. Gray points represent the non-DEGs.

Transcriptomes of EOMA cells were investigated according to methods mentioned above. In total, 15573 genes were identified. Firstly, to quality control, we analyzed the correlation of gene expression of biological replicates. R^2^ was greater than 0.98, indicating that the correlation of samples was ideal (Figure 1A). Furthermore, Principal component analysis (PCA) was performed to assess transcriptomic relationships among control and DHTS-treated groups based on the combination of PC1 and PC2 (Figure 1B). The largest distinction was 76.46%, between control and DHTS-treated samples (PC1), and the distinction among biological replicates was 7.04% (PC 2). These results illustrate clear differences between control samples versus DHTS-treated samples. As shown in Figure 1C, hierarchical clustering analysis revealed variations in gene expression between the two groups. To compare gene expression between the two groups, differential expression analysis was performed, and significant DEGs were acquired with the criteria of fold change > 1.5 and P < 0.05. Differential transcriptional analysis revealed differential expression of 2462 genes following treatment with DHTS for 24 h. Among them, 1620 genes were increased and 842 genes were decreased. The volcano plot in Figure 1D shows DEGs between two groups.

**Figure 2:**
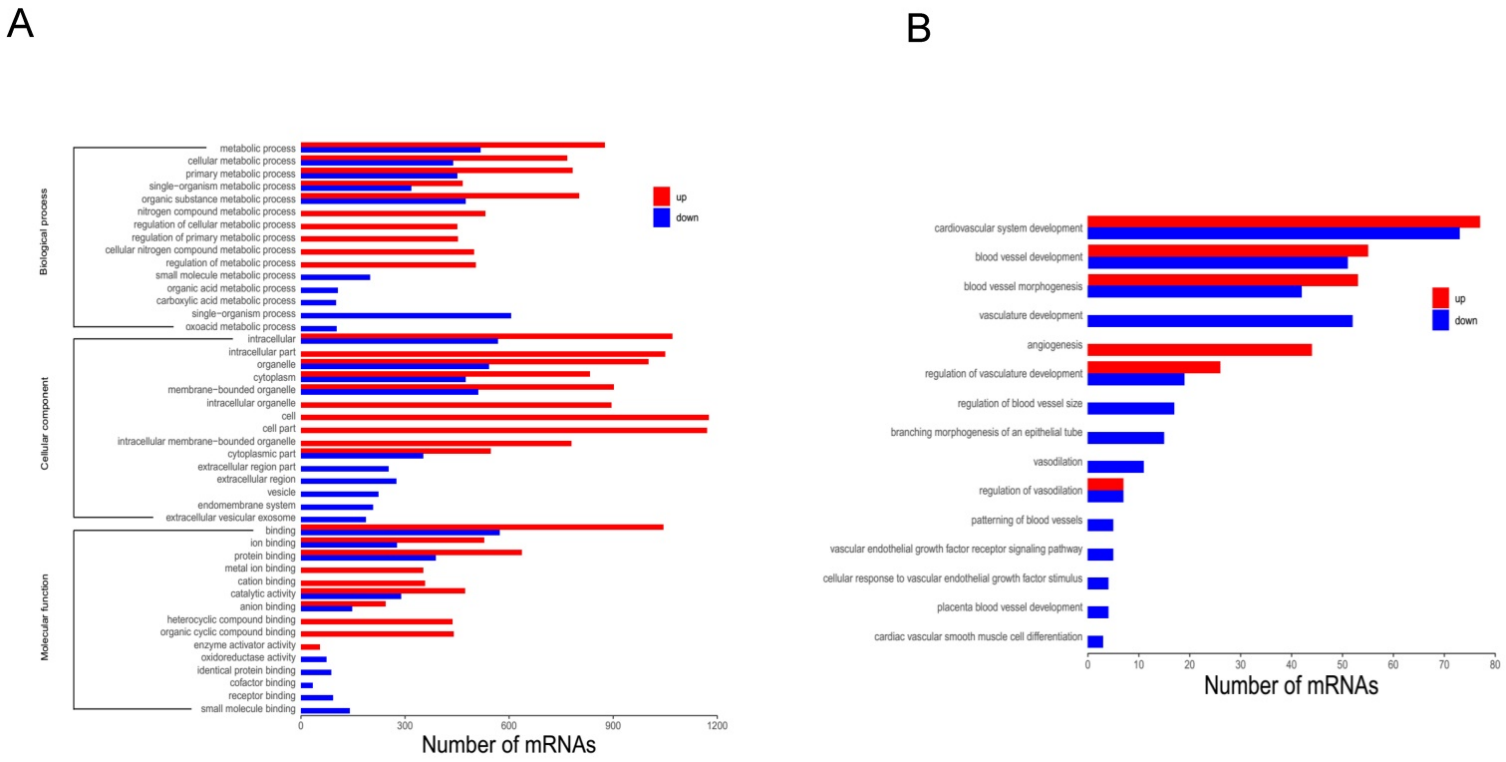
GO analysis of DEGs. **(A)** GO analysis of DEGs. Bars in red refer to up regulated paths, whereas the blue ones to the down regulated paths. Transcripts were grouped under three main categories: biological processes, cellular components and molecular function, indicated at the left of the figure. The X-axis represents the number of genes, and the Y-axis represents the path name. **(B)** Among BP category terms, the process related to vascular function. Bars in red refer to up regulated paths, whereas the blue ones to the down regulated function.

To further investigate functional differences in DEGs, GO analysis was employed. GO analysis is divided into three categories: biological process (BP), molecular function (MF), and cellular component (CC). Upon comparing DHTS-treated cells versus control cells, the most significant differences were observed in “metabolic process” (BP), “intracellular” (CC), and “binding” (MF), in addition to other processes (Figure 2A). Among BP category terms, we focused on the process of vascular function, and found that DHTS treatment impacted cardiovascular system development, blood vessel development, blood vessel morphogenesis, and other related processes. As shown in Figure 2B, red bars represent the activated function and blue ones represent the suppressed function. DHTS showed bidirectional regulatory effects on blood vessel development, blood vessel morphogenesis, regulation of vasculature development and so on. While DHTS showed significant down-regulation effects on vasculature development, branching morphogenesis of an epithelial tube, vascular endothelial growth factor receptor signaling pathways and so on.

**Figure 3:**
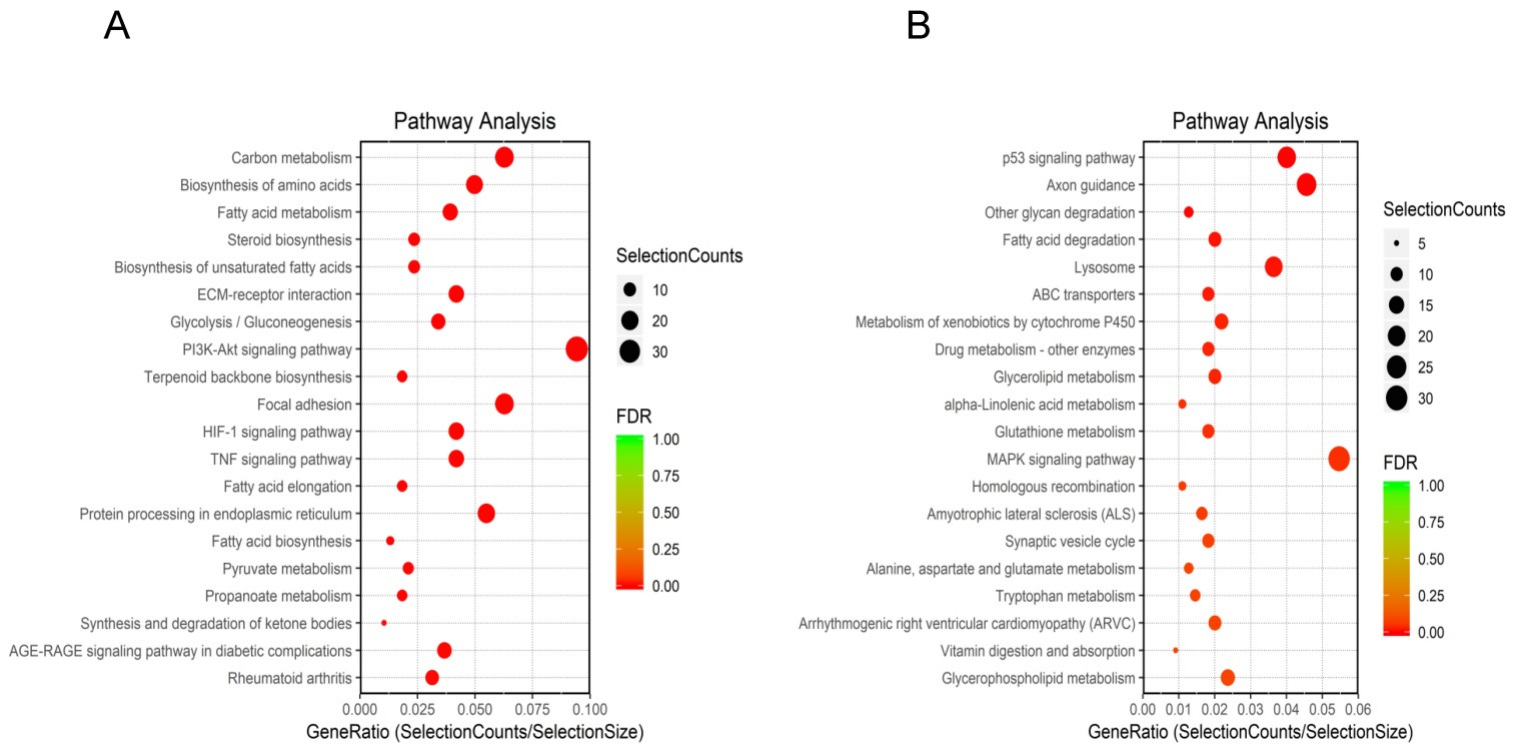
KEGG analysis of DEGs. **(A)** Top 20 downregulated pathways enrichment distribution of DEGs. The X-axis represents gene ratio, and the Y-axis represents the path name. The color represents FDR value, and less FDR value means greater intensiveness. The size of the point represents the number of DEGs. **(B)** Top 20 upregulated pathways enrichment distribution of DEGs. The X-axis represents gene ratio, and the Y-axis represents the path name. The color represents FDR value, and less FDR value means greater intensiveness. The size of the point represents the number of DEGs.

KEGG pathway analysis identified 127 enriched pathways. Among them, 71 pathways were inhibited (Figure 3A), while 56 were upregulated (Figure 3B). Downregulated and upregulated pathways with the highest enrichment scores were carbon metabolism and p53 signaling, respectively.

Then, based on bioinformatics results, we consulted related literatures. We considerd that HIF-1α pathway, one of the top 20 downregulated pathways, is worth to further study. Although the pathogenesis of IH remains unclear, there are several popular hypotheses about it, and hypoxia induction is one of these hypotheses. Much evidence indicated that IH is induced by intrauterine hypoxia 17. Coincidentally, the results of RNA-seq revealed significant downregulation of the HIF-1α signaling pathway following DHTS treatment. So, in the following study, we explored whether DHTS regulates HIF-1α and its mechanism.

### DHTS Downregulated HIF-1α Expression at the Post-Transcriptional Level

**Figure 4:**
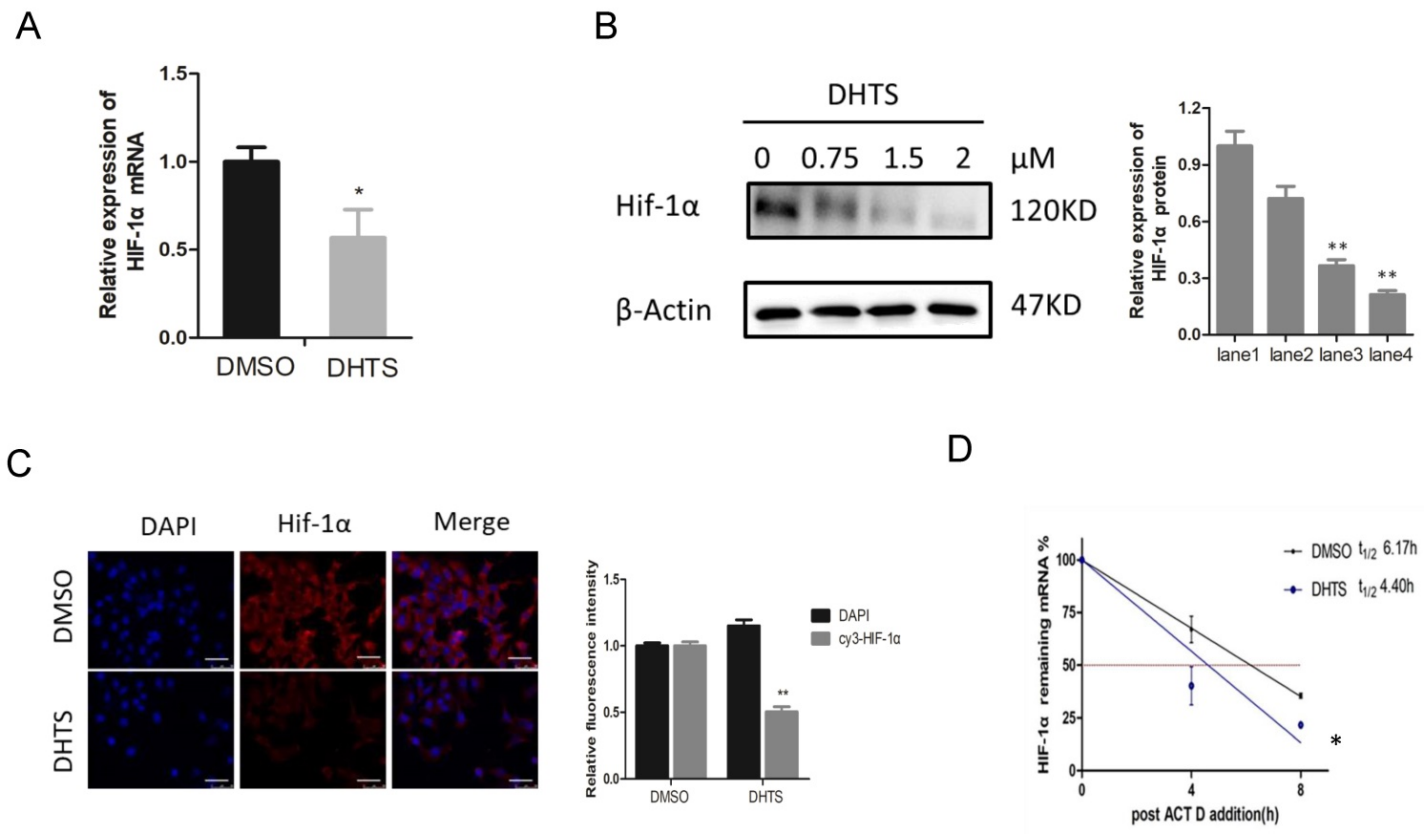
DHTS downregulated HIF-1α expression at the post-transcriptional level. (**A**) qRT-PCR reveals that compared with control group, HIF-1α mRNA in DHTS treated group was significantly decreased. ( *p < 0.05); (**B**) Western blot analysis. The lane1 is the control group. Compared with control group, DHTS could decrease the expression of HIF-1α protein. ( * compared with lane 1,p < 0.05; ** compared with lane1, p < 0.01); (**C**) Immunofluorescence results show that after 48 h of DHTS administration, HIF-1α protein expression was reduced compared with control group. Scale bars represent 50μm. The magnification was 400×. (** compared with control group, p < 0.01); (**D**) Stability of HIF-1α mRNA was detected, the remaining mRNA in control cells was relatively sustained after the addition of Act D within 8h, whereas it was significantly reduced in DHTS-treated cells. (*compared with control group, p < 0.05 ).

To clarify the mechanism by which DHTS acts on HIF-1α signaling pathway, we investigated mRNA and protein levels of HIF-1α. As shown in Figure 4A, after treatment with 2 μM DHTS for 24 h, the expression of HIF-1α mRNA was decreased in DHTS-treated group. After treatment with low (0.75 μM), medium (1.5 μM) and high (2 μM) concentrations of DHTS for 48 h, the expression of HIF-1α protein reduced in the DHTS-treated group compared with the control group. Moreover, with the increase of concentration, DHTS was more effective on HIF-1α protein inhibition (Figure 4B). Immunofluorescence results also showed that after 48 h of 2 μM DHTS administration, HIF-1α protein expression was reduced (Figure 4C). We also detected the stability of HIF-1α mRNA by examining the kinetics of the level of remaining HIF-1α mRNA to calculate its half-life. As shown in Figure 4D, the remaining mRNA in control cells was relatively sustained after the addition of Act D, whereas it was significantly reduced in DHTS-treated cells, indicating a significantly shorter half-life.

### DHTS Inhibited HuR-Associated HIF-1α Augmentation in Response to Hypoxia

**Figure 5:**
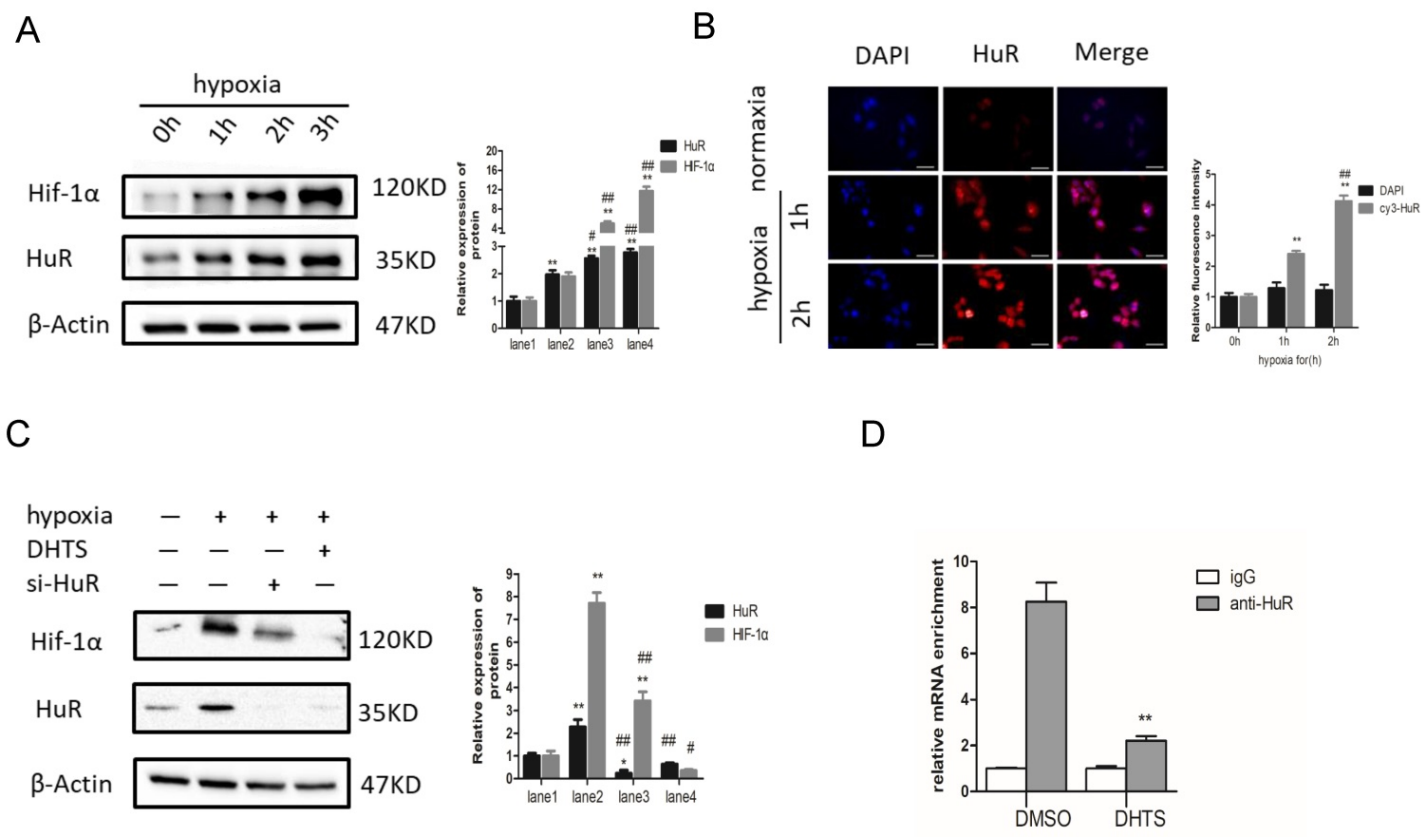
DHTS inhibited HuR-associated HIF-1α augmentation in response to hypoxia. (**A**) The lane1 is the control group. Western blot showed that, compared with the control group, HIF-1α and HuR expression was significantly increased in response to hypoxia. (*compared with 0h, p < 0.05; ** compared with 0h, p < 0.01; **#**compared with 1h, p < 0.05; **##** compared with 1h, p < 0.01); (**B**) Immunofluorescence results demonstrated augmentation of HuR and distribution to the cytoplasm of HuR in response to hypoxia. Scale bars represent 50μm. The magnification was 400×. (** compared with 0h, p < 0.01; **##** compared with 1h, p<0.01); (**C**)The hypoxic treatment maintained for 3h. The lane1 is the control group. Western blot showed that pretreatment with 2 μM DHTS for 24 h and si-HuR could both inhibit the hypoxia-induced increase in HIF-1α expression. DHTS could more strongly inhibit HIF-1α overexpression than si-HuR. (* compared with lane 1, p < 0.05; ** compared with lane1, p < 0.01; **#**compared with lane2, p < 0.05; **##** compared with lane2, p < 0.01). (**D**) RNA-IP indicated that HuR could directly bind to HIF-1α mRNA in EOMA cells, and DHTS interfered with the binding of HuR and HIF-1α mRNA. ( ** compared with DMSO group, p < 0.01).

Since the stability of HIF-1α mRNA and the expression of HIF-1α protein were decreased significantly, we speculated that DHTS may regulate HIF-1α at the post-transcriptional level. It is well known that the ELAVL family is a class of RNA-binding proteins regulating mRNA stability and translation process in response to stimuli such like hypoxia. And post-transcriptional regulation of HIF-1α by HuR (ELAVL1) has already been confirmed in cell models of other diseases 18. To study the regulation of HIF-1α by HuR in response to hypoxia in EOMA cell model, we detected HIF-1α and HuR protein expression after hypoxic treatment for 0 h, 1 h, 2 h, and 3 h, separately. HIF-1α and HuR protein expression was significantly increased 2-3 h after hypoxia (Figure 5A). After hypoxic treatment for 1 h, the expression of HuR protein was statistically higher compared with normoxia group. While for HIF-1α protein, after hypoxic treatment for 2 h, the protein expression between hypoxia group and normoxia group showed statistically different. It may indicate that the overexpression of HIF-1α protein is following after the increase of HuR protein. Inactive HuR protein mainly expresses in nucleus, and the function of mRNA stability regulation of HuR depends on its nucleus-plasm shuttle. Therefore, we detected the expression and localization of HuR by immunofluorescence. Immunofluorescence results demonstrated augmentation of HuR expression within 2 h of hypoxia. More importantly, HuR was localized in nuclear compartments in normoxia cells, but was apparently distributed to the cytoplasm of hypoxia-exposed cells (Figure 5B).

To further investigate the role of HuR in HIF-1α augmentation in response to hypoxia, we used small interfering RNA (siRNA) to block HuR expression. The results showed that in HuR knocked down cells, the augmentation of HIF-1α protein is significantly inhibited compared with it in negative control ones in response to 3h-hypoxia (Figure 5C). It indicates that hypoxia-induced overexpression of HIF-1α protein depends on the function of HuR in EOMA cells.

On the other hand, we pretreated EOMA cells with 2 μM DHTS for 24 h before 3h hypoxic exposure. The western blot results showed that DHTS could counteract the hypoxia-induced augmentation in HIF-1α expression (Figure 5C).

Moreover, we performed RNA-IP to detect the combination of HuR and HIF-1α mRNA in EOMA cells, as well as the effects of DHTS on this combination. Using an HuR antibody, precipitation levels of HIF-1α mRNA were detected by PCR with reverse transcription. The igG group was used as the negative control to exclude the non-specific binding, and the value of HIF-1α mRNA binding to HuR in each group was showing in Figure 5D. The results indicated that HuR could directly bind to HIF-1α mRNA in EOMA cells, and DHTS interfered with the binding of HuR and HIF-1α mRNA.

## Discussion

IH occurs as a result of abnormal angiogenesis.The pathogenesis of IH has not been completely elucidated. There are several popular hypotheses about its pathogenesis. First of all, hypoxia is considered as an inducer of IH. Under hypoxic environment, as a key transcription factor response to hypoxia stimulation, upregulated HIF-1α could activate pro-angiogenesis mediators such as VEGF and VEGFR 19-21. Secondly, a theory holds that IH originated from endothelial progenitor cells, which could differentiate into endothelium, adipocytes and pericytes under internal and external stimuli such as tissue ischemia. In addition, the theory of placental origin holds that placental cells may cause embolism during pregnancy and delivery, which is the cause of IH in embolic tissues of the newborn. 22-23.

In this study, we used EOMA cell line as a model to investigate the mechanism of DHTS on endothelial cells, which represents the possible mechanism of DHTS on IH. We used a transcriptomics approach to evaluate the mechanism by which DHTS inhibits the proliferation of EOMA cells. According to the clues provided by bioinformatic results and the published literatures, we focused on HIF-1α for further research 24. Epidemiological investigations have shown that the incidence of IH is closely related to the state of hypoxemia during pregnancy 25. In low-birthweight premature infants, a group with high incidence of IH, the incidence of intrauterine hypoxia is also three times than that of in normal infants 26. Analysis of IH tissue samples revealed abnormally high expression levels of HIF-1α and vascular endothelial growth factor compared with normal tissues 27. It is well known that HIF-1α is a master transcription factor in the process of angiogenesis through stimulating vascular endothelial growth factor 28. Moreover, the first-line treatment propranolol, has been demonstrated to exert its suppressive effect on hemangioma through the HIF-1α-VEGF-A angiogenesis axis 29. Our previous work validated that DHTS could effectively inhibit the proliferation and angiogenesis of EOMA cells 8. In this study, we revealed DHTS could depress HIF-1α expression levels via a post-transcriptional process.

**Figure 6:**
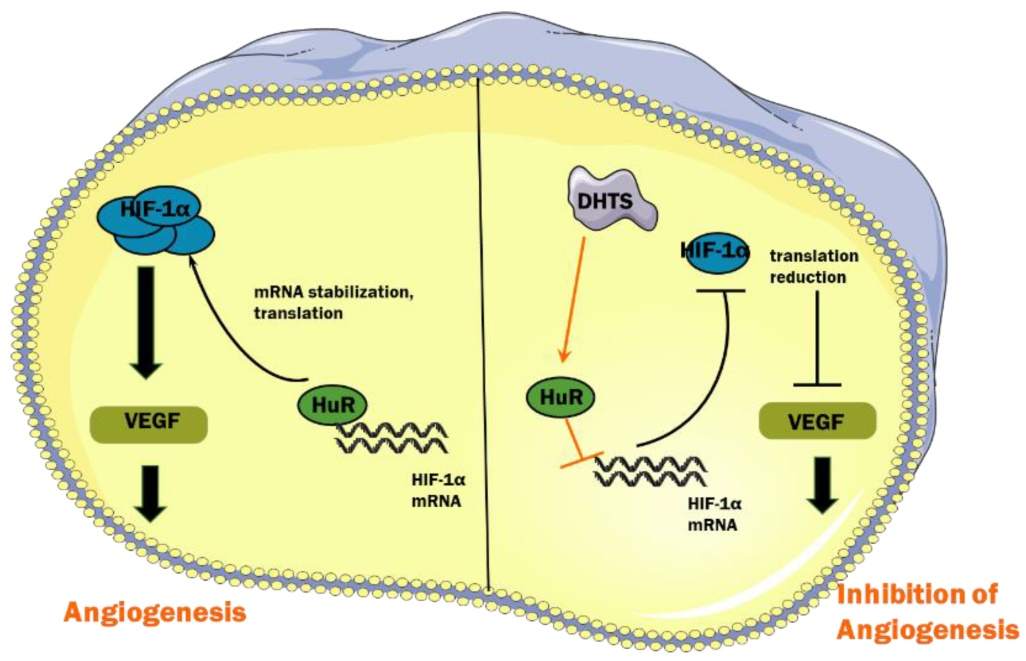
Proposed working model of DHTS on HIF-1α signaling pathway in EOMA cells.

Under hypoxic conditions, hydroxylation of HIF-1α protein mediated by von Hippel-Lindau protein is suppressed, which enhances the protein stability of HIF-1α 30. In addition, the interaction of RNA-binding proteins and HIF-1α mRNA enhances its stability and improves its translation efficiency, thus increasing the total amount of HIF-1α protein 31. RNA-binding proteins are a class of proteins that can bind and splice target RNA, regulate mRNA stability and translation process, and induce cell differentiation, apoptosis, or proliferation in response to different stimuli and microenvironments 32. HuR, encoded by the ELAVL1 (embryonic lethal, abnormal vision Drosophila-like 1), is an important RNA-binding protein that plays a major role in hypoxic response mechanisms 33. Its molecular weight is 36 kD. HuR has three RNA recognition motifs, which can selectively combine with adenylate-uridine acid-rich elements in untranslated regions to enhance mRNA stability and carry it to the cytoplasm, thereby promoting translation 34,35. It has been reported that HuR binds to HIF-1α mRNA to enhance its stability and promote translation efficiency, resulting in an increase in the expression of HIF-1α protein 18. In our study, we found that in EOMA cells, DHTS could decrease the stability of HIF-1α mRNA as well as the expression of HIF-1α protein. RNA-IP identified the direct binding of HuR with HIF-1α mRNA in EOMA cells, and the binding was impaired by DHTS. These results suggest that via acting on HuR, DHTS negatively regulates the post-transcriptional process of HIF-1α, thereby inhibiting the proliferation and angiogenesis of EOMA cells. The proposed working model of DHTS on HIF-1α signaling pathway in EOMA cells is shown as Figure 6.

It is worth to note that regulating the interaction with HuR may not be the only pathway by which DHTS inhibits HIF-1α protein expression. In figure 5C, compared to HuR-knocked down, DHTS showed stronger effect on inhibiting HIF-1α protein overexpression in response to hypoxia. It has been reported that in human gastric cancer cell model, DHTS could inhibit HIF-1α expression by suppressing its protein accumulation 36. And DHTS could inhibit HIF-1a protein synthesis through downregulating the activity of mTOR/p70S6K/4E-BP1 and MEK/ERK pathways 37. These suggests that in EOMA cells, in addition to interfering with the posttranscriptional process of HIF-1α via regulating HuR, other mechanisms of DHTS remain to be explored.

In this study, we proposed for the first time that HuR participates in the process of EOMA cells proliferation, which indicated that HuR may play a role in the occurrence of hemangioma. Studies have found that HuR mediates pathological processes such as tumor development, cardiovascular disease, and vascular endothelial abnormalities 38. For example, in pancreatic ductal adenocarcinoma cells, under hypoxic stress, HuR stabilizes the mRNA of a hypoxia-inducible and pro-oncogenic kinase PIM1, resulting in its protein overexpression, thereby promoting tumor cells growth 39. It was also reported that after knocked-out HuR, mice exhibited reduced revascularization and tumor angiogenesis, which means that lower expression of HuR could attenuate blood flow and tumor growth 40. In our study, a siRNA specific to HuR was used to validate its role in inducing HIF-1α overexpression under hypoxia. When HuR was knocked down by siRNA, overexpression of HIF-1α protein was strongly inhibited in response to hypoxia. It suggests that as an important member of RNA-binding proteins, HuR may be worthy of further study in terms of pathogenesis and treatment in hemangioma.

## Conclusions

DHTS could inhibit the proliferation and angiogenesis of EOMA cells through the HIF-1α signaling pathway. Moreover, interfering with the interaction between RNA binding protein HuR and HIF-1α mRNA may be one of its mechanisms.

## Abbreviations

IH: Infantile hemangioma
DHTS: 15,16-dihydrotanshinone I
HIF-1α: hypoxia-inducible factor 1 alpha
FBS: fetal bovine serum
DMSO: dimethyl sulfoxide
IC50: half maximal inhibitory concentration
GO: Gene Ontology
DEG: differentially expressed gene
KEGG: Kyoto Encyclopedia of Genes and Genomes
PCR: Polymerase Chain Reaction
Act D: actinomycin D
PBS: phosphate-buffered saline
SDS: sodium dodecyl sulfate
DAPI: 4',6-diamidino-2-phenylindole
RNA-IP: RNA immunoprecipitation
PCA: Principal component analysis
BP: biological process
MF: molecular function
CC: cellular component
siRNA: small interfering RNA
ELAVL1: embryonic lethal, abnormal vision Drosophila-like 1
